# Phase 3 Trial of Oral Anti‐Amyloid Agent ALZ‐801/Valiltramiprosate in APOE4/4 Homozygotes with Early Alzheimer’s Disease: Baseline Characteristics and Prevalence of Comorbid Cerebral Amyloid Angiopathy

**DOI:** 10.1002/alz.095679

**Published:** 2025-01-09

**Authors:** Aidan Power, David Watson, Susan Abushakra, Emer MacSweeney, Anton P. Porsteinsson, Marwan N. Sabbagh, Earvin Liang, Rosalind McLaine, Mercè Boada, Jón Snædal, Susan Flint, Winnie Pak, John A. Hey, Martin Tolar

**Affiliations:** ^1^ Alzheon, Inc., Framingham, MA USA; ^2^ Alzheimer’s Research and Treatment Center, Wellington, FL USA; ^3^ Re‐Cognition Health, London United Kingdom; ^4^ University of Rochester School of Medicine and Dentistry, Rochester, NY USA; ^5^ Barrow Neurological Institute, Phoenix, AZ USA; ^6^ Alzheon Inc., Framingham, MA USA; ^7^ Ace Alzheimer Center Barcelona – International University of Catalunya (UIC), Barcelona Spain; ^8^ Landspitali University Hospital, Reykjavik Iceland; ^9^ Alzheon, Framingham, MA USA

## Abstract

**Background:**

ALZ‐801/valiltramiprosate, an oral small molecule inhibitor of amyloid oligomer formation, is being evaluated in a Phase 3 trial in APOE4/4 Early AD subjects (APOLLOE4). Topline results are expected in 3Q 2024. APOE4 is a major risk factor for Alzheimer’s disease (AD) and cerebral amyloid angiopathy (CAA) with a gene‐dose effect. APOE4/4 subjects with CAA are at higher risk of amyloid related imaging abnormalities (ARIA) with anti‐amyloid antibodies, including ARIA‐E (effusion/edema) and ARIA‐H [lobar microhemorrhages (MH), macrohemorrhages, and superficial siderosis (SS)]. APOE4 carriers are also at higher risk of hyperlipidemia and cardiovascular disease (CVD). Baseline prevalence of CAA and CVD risk factors were analyzed.

**Method:**

This Phase 3 trial (NCT04770220) enrolled 325 APOE4/4 Early AD subjects (MMSE ≥22, 50‐80 years); 313 had baseline MRIs with central readings. Subjects with MH and SS were enrolled; ARIA‐E was exclusionary. Subjects with CAA (>4 MH, any siderosis, any macrohemorrhage) were compared to rest of population.

**Result:**

Study population was 51% female, age 69 years, MMSE 26, 65%/MCI, 82% Caucasians. CAA group included 47/313 subjects (15%) of study, was mostly male (70% vs 45%), older (71 vs. 68 years, p = 0.004), more advanced (MMSE 25 vs 26, p = 0.018; ADAS‐cog 27 vs. 23, p = 0.002) with more severe deep WMD (p = 0.015). Both groups had similar prevalence of hypertension, diabetes, obesity, hyperlipidemia, and statin use but with higher coronary artery disease prevalence (17% vs. 8%, p = 0.049) and anti‐platelets use (38% vs 22%, p = 0.026) in the CAA group. No anticoagulants were allowed.

**Conclusion:**

ALZ‐801 showed no ARIA‐E in prior AD studies allowing its evaluation in the high‐risk APOE4/4 homozygotes. This pivotal APOLLOE4 Phase 3 trial of ALZ‐801 enrolled homozygous APOE4/4 subjects with higher CAA burden than anti‐amyloid antibody trials. The APOE4/4 CAA subjects were older with more advanced AD and higher rates of CAD and antiplatelets use than non‐CAA subjects, which may increase brain edema and microhemorrhage risk, requiring heightened vigilance with anti‐amyloid antibodies. If this Phase 3 trial is positive, ALZ‐801 could become a potentially safer alternative to anti‐amyloid antibodies for this population. The Phase 3 results are expected in 3Q 2024.

